# Promotion of Ionic Conductivity of PEO-Based Solid Electrolyte Using Ultrasonic Vibration

**DOI:** 10.3390/polym12091889

**Published:** 2020-08-21

**Authors:** Hui Wang, Xiaodong Cui, Cong Zhang, Huang Gao, Wei Du, Yizhe Chen

**Affiliations:** 1 Hubei Key Laboratory of Advanced Technology for Automotive Components, Wuhan University of Technology, Wuhan 430070, China; huiwang@whut.edu.cn (H.W.); 823416242@whut.edu.cn (X.C.); congzhango@whut.edu.cn (C.Z.); wei.du@whut.edu.cn (W.D.); 2 Hubei Collaborative Innovation Center for Automotive Components Technology, Wuhan 430070, China; yzchen@whut.edu.cn; 3 State Key Laboratory of Material Processing and Die & Mould Technology, School of Materials Science and Engineering, Huazhong University of Science and Technology, Wuhan 430074, China

**Keywords:** solid electrolyte, ultrasonic vibration, polyethylene oxide, ionic conductivity, improvement, crystallinity

## Abstract

All solid-state lithium-ion batteries based on polymer electrolytes have higher safety and energy density, but the low conductivity of lithium ion restricts its application. This study proposes a new method to promote the ionic conductivity of polyethylene oxide (PEO)-based solid electrolytes. In this method, the PEO-based solid electrolyte was first prepared by casting, and then power ultrasound was exerted on the electrolyte by a sandwich structure to modify the electrolyte structure. Through analysis of the performance and microstructure of the electrolyte, it was found that the ultrasonic treatment increased the ionic conductivity by 78%, improved tensile strength and plastic deformation ability, but did not affect the thermal stability and the chemical composition. The ultrasonic vibration, exerting high energy to the solid electrolyte through high-frequency vibration, broke PEO grains and melted them with the frictional heat at boundary. Due to the slight melting and fast solidifying produced by the pulsed ultrasonic treatment, the crystallization was suppressed. The crystallinity was thus reduced by 6.2%, which increased the migration channels of lithium ions and reduced the tortuosity effect. Furthermore, the ultrasonic vibration compressed the electrolyte to produce plastic flow of the material, which made the electrolyte structure more compact. The density of ethylene oxide (EO) units thus increased in the amorphous phase, providing multiple electron-donor coordination sites for the Li^+^. The hopping distance of the ion between donors decreased, which also facilitated the migration. In addition, the mechanical performance of the electrolyte membrane improved. This study provides a reference for the improvement of polymer based all-solid-state batteries.

## 1. Introduction

In recent years, with the advancement of new energy vehicles, the market has put forward new requirements for batteries. As the core component of electric vehicles, power batteries will encounter new opportunities and challenges. In the future, the energy density of the power batteries of electric vehicles must reach at least 500 W·h/kg, but the energy density of large-scale practical lithium-ion batteries is far from this goal. Compared with traditional lithium-ion batteries, the all-solid-state lithium ion battery has better safety and higher energy density [1], and are expected to be used in electric vehicles, and therefore they have attracted widespread attention.

Compared with the liquid electrolyte, the solid electrolyte has poor conductivity of lithium ions, and the conductivity is usually only 10^−5^ S/cm, while the liquid electrolyte ion conductivity can reach 10^−2^ S/cm. The migration of lithium ions in the solid electrolyte is weaker than that in the liquid electrolyte [2,3,4,5,6,7]. The polyethylene oxide (PEO)-based solid electrolyte is the most widely studied in polymer based all-solid-state lithium batteries. PEO is a semi-crystalline polymer. Generally, lithium ion transport in a PEO-based solid electrolyte is derived from segmental movement that can be accelerated in the amorphous PEO compared with that in the crystalline PEO [8]. Therefore, an effective way to increase the ionic conductivity of the PEO-based electrolyte is to reduce its crystallinity and increase the proportion of the amorphous phase. 

At present, the main methods for reducing the crystallinity include plasticizers, ionic liquids, inorganic fillers and copolymers. Chintapalli S et al. [9] studied the effect of the plasticizer ethylene carbonate (EC) and propylene carbonate (PC) on the high molecular weight PEO_9_-LiCF_3_-SO_3_ system, and found that the addition of the plasticizer significantly improved the ionic conductivity of the electrolyte at room temperature. Kumar et al. [10] added 1-Ethyl-3-methy limidazolium trifluoromethane-sulfonate (EMITF) ionic liquid to the PEO-based electrolyte system, and found that the organic cation EMI^+^ can interact with the PEO segment, increase the amorphous phase ratio, and improve the lithium ion transport capacity. Scrosati et al. [11] studied the influence of different inorganic ceramic fillers on the performance of the solid polymer electrolyte PEO-LiCF_3_SO_3_ system, and found that the addition of inorganic ceramic fillers can effectively improve the interface stability of the electrolyte and the lithium metal electrode, and enhance the electrolyte’s overall transport properties. Tan SM et al. [12] studied the effect of nano-${\mathrm{MnO}}_{2}$ filler on the ionic conductivity of the plasticized polymer electrolyte PMMA-PEO-LiClO_4_-EC system. By optimizing the composition of the polymer, salt, plasticizer and filler, the ionic conductivity of the electrolyte can reach 10^−3^ S/cm. K. Nairn et al. [13] added the ceramic solid electrolyte ${\mathrm{Li}}_{1.3}{\mathrm{Al}}_{0.3}{\mathrm{Ti}}_{1.7}{{(\mathrm{PO}}_{4})}_{3}$ into a pure amorphous copolymer electrolyte polyethylene glycol(PEG)-LiCF_3_SO_3_ to prepare an organic–inorganic solid electrolyte. When the inorganic filler was 66 wt.% and the temperature was 40 °C, the composite electrolyte had an ionic conductivity of (1.9 ± 0.2) × 10^−4^ S/cm. Xiao-Yuan Yu et al. [14] prepared a new type of PEO/poly propylene carbonate (PPC) solid electrolyte by blending PEO with poly propylene carbonate (PPC), and found that the addition of PPC can effectively reduce the glass transition temperature Tg and crystallinity of the PEO and improve the ionic conductivity of the electrolyte. Niitani T et al. [15] applied a block copolymer consisting of PEO and polystyrene (PS) to prepare a LiClO_4_-contained solid polymer electrolyte with high ionic conductivity at room temperature.

Power ultrasound, an interesting processing method for polymer materials, produces thermal and mechanical changes in the materials by low-amplitude and high-frequency vibration [16,17]. Related research has shown that the ultrasound can produce high-frequency vibration effects on the blend melt, and can effectively promote the mutual diffusion and fusion of molecular chains of different polymers. Ultrasonic treatment enhances the interaction between molecules, promotes chemical bonds between different polymers without using any chemical catalysts, induces in-situ copolymer at the interface of polymer blends, causes micromechanical mixing, and improves the interface bond of phases [18,19]. Madhusudan Bera et al. [20] found that the ultrasound promoted the formation of covalent bonds, thereby producing macromolecular structures. The ultrasonic frequency was 15–120 kHz in actual use. The mechanical vibration preferentially generated heat at the interface through the friction effect, and therefore the interface bonding strength can reach 100% of the matrix [21]. Fernandez Villegas et al. [22] proved that the high-frequency vibration of the ultrasound can generate ultra-short heating at the interface, causing the thermoplastics to melt, and thus two pieces can be joined together under pressure. Chu et al. [23] used differential scanning calorimetry (DSC) to study the crystallinity of samples prepared by the ultrasonic vibration–assisted automated fiber placement (UAFP) and hot pressing. The results showed that the crystallinity of the UAFP sample was only 38.5%, while the crystallinity of the hot-pressed sample was 49.2%.

In published reports, there is no study on the application of ultrasonic vibration for the improvement of the ionic conductivity of the solid polymer electrolyte. If the polymer electrolyte is subjected to ultrasound, high-frequency vibration breaks the electrolyte grains, increasing the ratio of the amorphous phase, and thereby improves the lithium ion conductivity. In this study, a PEO-based solid electrolyte was first prepared by casting, and then power ultrasound was exerted on the electrolyte to modify the structure. Through tests on the structure and performance of the electrolyte, the effect of the ultrasonic vibration was analyzed.

This paper is organized as follows. Section 2 describes the materials and ultrasonic treatment method. Section 3 gives the results and discussion. Finally, Section 4 summarizes our conclusions.

## 2. Materials and Methods 

### 2.1. Materials

**Figure 1:**
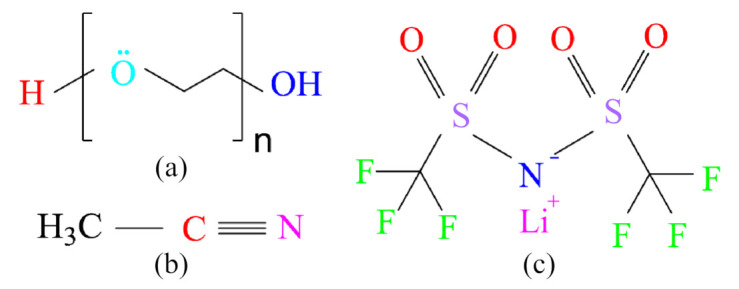
Structural formulas of (**a**) polyethylene oxide (PEO), (**b**) acetonitrile, and (**c**) lithium bis ((trifluoromethyl) sulfonyl) azanide (LiTFSI).

The resin-matrix material for the solid polymer electrolyte was PEO (Mw = $10^{6}$; Ryoji Organic Chemical, Shanghai, China). The melting temperature is 65 °C, and the density is 1.15–1.22 g/cm^3^. The structural formula is shown in Figure 1a. The ether group C–O–C is the function group, which provides electron-donor coordination site for the Li^+^ ion. In addition, the end group C–OH is also involved in the molecule. Acetonitrile (ACN, 99.8%; Tianjin Beilian Fine Chemicals Development Co., Ltd., Tianjin, China), and lithium bis ((trifluoromethyl) sulfonyl) azanide (LiTFSI, 99%; Shanghai Aichun Biological Technology, Shanghai, China) were used for the solvent and lithium salt, respectively. The density of the lithium salt is 1.334 g/cm^3^. The structural formulas are shown in Figure 1. The group C≡N is the characteristic group, and can be used to identify the presence of the acetonitrile. The sulfonyl group O=S=O and the trifluoromethyl group CF_3_ are both characteristic groups of the salt LiTFSI.

### 2.2. Experimental Method

#### 2.2.1. Preparation of Polymer Solid Electrolyte Membrane

**Figure 2:**
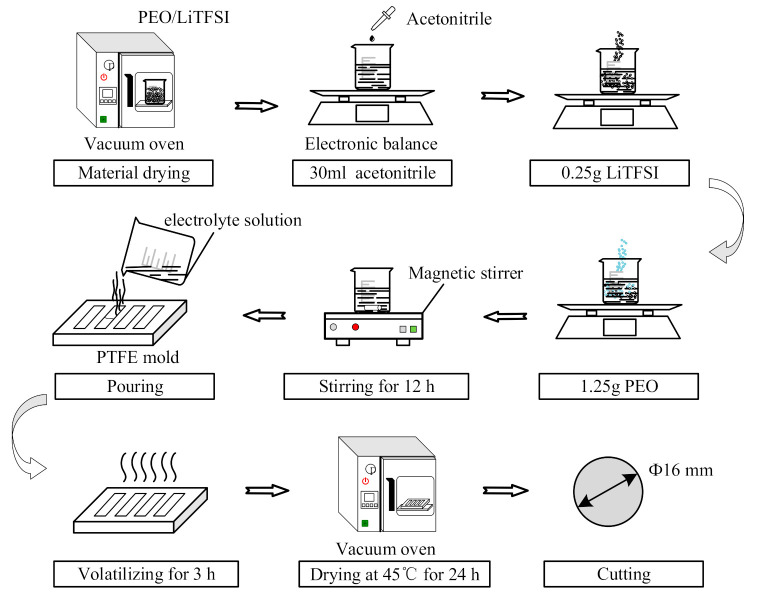
Preparation process of the solid electrolyte membrane.

**Figure 3:**
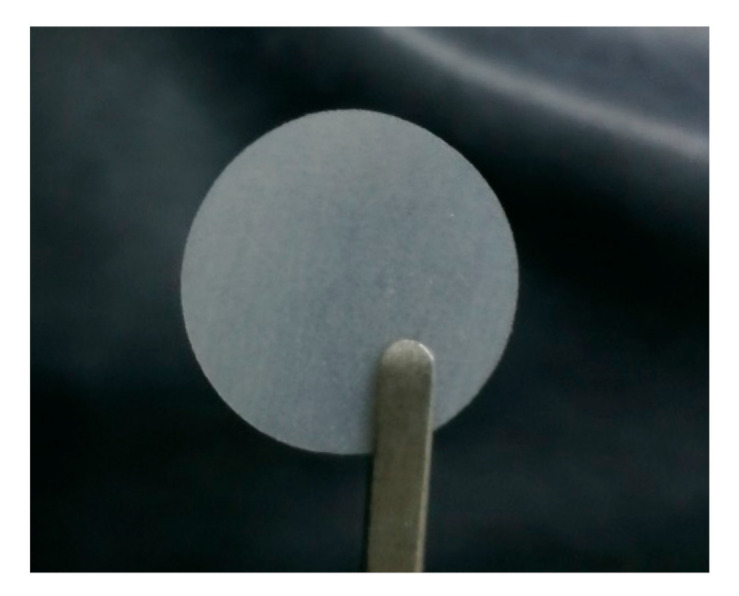
Cut pieces of the electrolyte.

The preparation process of the solid electrolyte membrane is shown in Figure 2. The PEO powder and LiTFSI were dried in a vacuum oven at 60 °C for 24 h to remove residual moisture, and then these materials were put into a glove box (Super, Mikrouna, Shanghai, China). To obtain an excellent solid polymer electrolyte (SPE), the ratio of ethylene oxide (EO):Li=18:1 is required, and thus 1.25 g PEO and 0.25 g LiTFSI were weighed with a precision electronic balance (8086, Dongguan DiHeng instrument, Dongguan, China). Then, 30 mL acetonitrile was poured into a clean, dry beaker. Firstly, the LiTFSI was added to the beaker, and the solution was stirred magnetically with a magnetic stirrer (ZGCJ-3A, Shanghai ZiGui instrument, Shanghai, China). After the LiTFSI was dissolved completely, the PEO was added slowly. Subsequently, the beaker was placed on the magnetic stirrer, with which the solution was stirred for 12 h. After that, the solution appeared transparent and homogeneous. The solution was quickly poured into a polytetrafluoroethylene (PTFE) mold to form a membrane. The solution flowed naturally in the cavity until it was filled up. The mold was kept in the glove box for 3 h to eliminate most acetonitrile solvent, and then the mold was dried in a vacuum oven (DZF-6020B, Changzhou EnPei instrument Co. Ltd., Changzhou, China) at 45 °C for 24 h. The electrolyte membrane was removed with a tweezer, and was cut into circular pieces of 16 mm in diameter, as shown in Figure 3. The pieces were kept in the glove box for later use.

#### 2.2.2. Ultrasonic Treatment of the Solid Electrolyte

**Figure 4:**
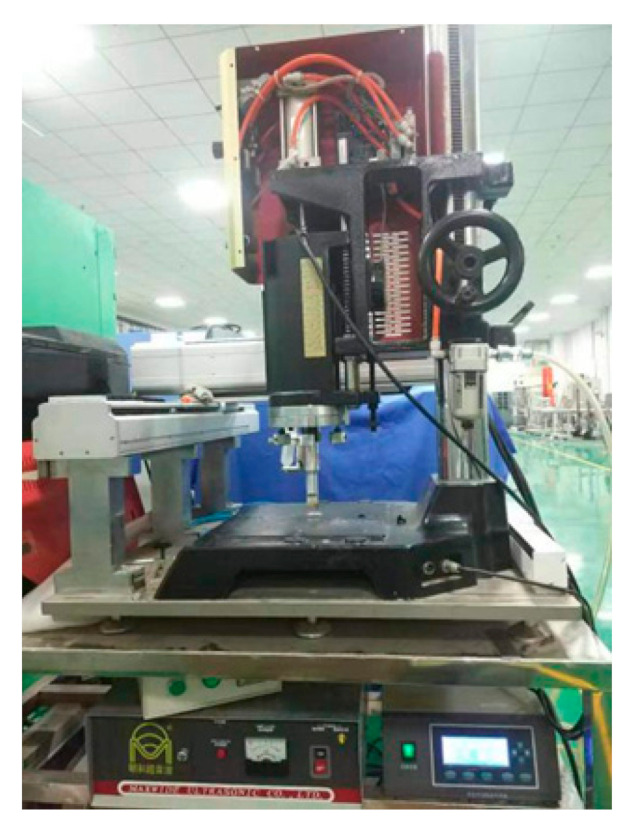
Experiment platform for the ultrasonic treatment.

In this experiment, the Taiwan MAXWIDE^®^ ME-1800 ultrasonic platform was applied, as shown in Figure 4. The device is a vertical structure, and is mainly composed of an ultrasonic generator, an ultrasonic transducer, a horn and a sonotrode. The transducer, horn and sonotrode are mounted on a pneumatic press machine, and can be moved up and down along a vertical guide-bar. The maximum pressure is 0.8 MPa. When the device is in operation, the ultrasonic generator converts civilian electricity of 220 V and 50 Hz into high-frequency alternating current (AC) electrical signals to match with the ultrasonic transducer. Then the transducer converts the high-frequency electrical signals into mechanical vibration. The horn, made of steel, amplifies the vibration. The sonotrode is of a cylinder type, which is made of 7075 aviation aluminum alloy. It is tightly fixed to the horn to concentrate the energy of the ultrasonic system on a relatively small area. 

**Figure 5:**
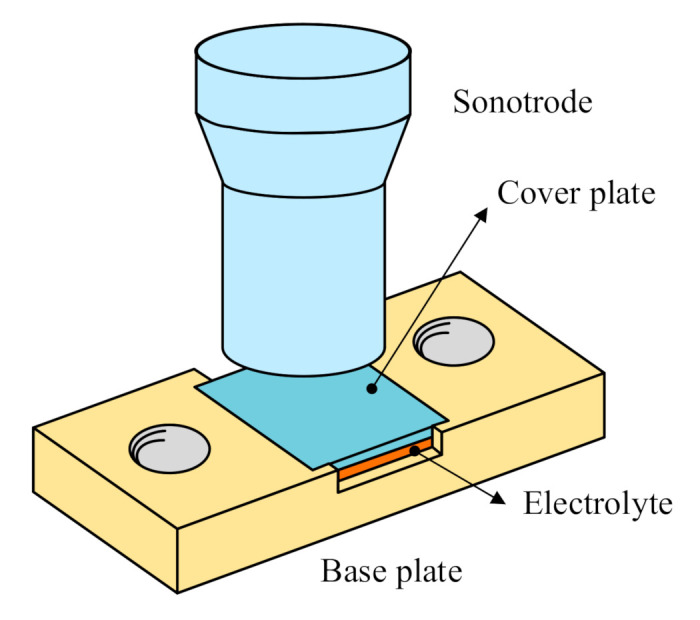
Schematic of the ultrasonic treatment.

Ultrasonic vibration was applied to modify the microstructure of the electrolyte. As shown in Figure 5, we used a sandwich structure, which included a cover plate, the electrolyte, and a base plate. The base plate, made of 7075 aluminum alloy, was fixed on the experimental platform to prevent the tearing of the electrolyte membrane due to the horizontal slip during the ultrasonic treatment. In order to apply uniform force on the electrolyte, a smooth plate was covered on the electrolyte membrane. The cover plate should not be too thick, otherwise it will affect the transmission of the ultrasonic vibration. It should not be too thin, otherwise the plate deforms easily due to the low rigidity during the application of the vibration, leading to inhomogeneous force on electrolyte membrane. In this study, a 20 × 20 × 2 mm carbon fiber reinforced plastics (CFRP) laminate was used as the cover plate. According to orthogonal experimental design, the ultrasonic frequency used was 15 kHz, the pressure was 0.24 MPa, the amplitude was 40 μm, and the vibration time was 32 s. During the vibration, an intermittent mode was applied to avoid electrolyte ablation caused by the ultrasonic overheating. In one cycle of the mode, the ultrasonic operated for 1 s, and suspended for 3 s. The solid electrolyte membrane without the ultrasonic treatment was recorded as Group A, and the solid electrolyte film treated by the ultrasonic vibration was recorded as Group B.

### 2.3. Characterization

#### 2.3.1. AC Impedance

The SPE was measured by an electrochemical workstation, and the ionic conductivity was then obtained by the analysis of the measured AC impedance spectra. The test instrument used was the CHI760E electrochemical workstation (Shanghai Chenhua Instrument, Shanghai, China). The structure of stainless steel (SS)/SPE/SS was applied for the test. The sweep frequency range was 0.1~10^6^ Hz, and the test temperature was from 25 °C to 70 °C.

#### 2.3.2. Direct Current (DC) Polarization

The conductivity was analyzed by the DC polarization method. A good solid electrolyte should have enough electronic resistivity, otherwise it will short circuit inside the battery. The test instrument used was the BTS4000 analyzer (NEWARE, Shenzhen, China). The structure of SS/SPE/SS was applied. The test voltage was 3 V, and the test time was 320 min.

#### 2.3.3. Linear Sweep Voltammetry (LSV)

The electrochemical window of the electrolyte was determined by the LSV test. The test instrument used was the CHI760E electrochemical workstation. The structure of Li/SPE/SS was applied. The initial voltage was 2.9 V, the termination voltage was 6 V, and the scanning frequency was 5 × 10^4^ Hz.

#### 2.3.4. Scanning Electron Microscope (SEM)

The microstructure of the electrolyte was observed by the JEM-7500F electron microscope (JEOL, Tokyo, Japan). Before the observation, the electrolyte was fractured in liquid nitrogen. After platinum coating, the fracture surface was observed.

#### 2.3.5. Mechanical Performance Test

Mechanical property is an important index to evaluate the solid electrolyte. Excellent mechanical property can effectively inhibit the growth of lithium dendrites and ensure safety during cycles. In this study, the Instron1341 electro-hydraulic servo testing machine (Instron, High Wycombe, UK) was used to test the mechanical property of the solid electrolyte. 

#### 2.3.6. Thermogravimetric Analysis (TGA)

Thermogravimetric analysis was used to test the thermal stability of the electrolyte. The test instrument used was the TGA 5500 analyzer (TA, New Castle, DE, USA). The sample used for the analysis was cut into 3 mm^2^. The temperature rise rate was set as 10 °C/min, and the temperature range was 0~800 °C.

#### 2.3.7. Fourier Transform Infrared (FTIR) Spectroscopy

In this study, the Nicolet 6700 infrared spectrometer (Thermo Fisher Scientific, Waltham, MA, USA) was used for the test of FTIR spectra. The wave number was from 65 cm^−1^ to 4000 cm^−1^.

#### 2.3.8. X-ray Diffraction (XRD)

An X-ray diffractometer was used to examine the crystallinity, and the XRD pattern was analyzed to calculate the change in the crystallinity of the electrolytes with and without the ultrasonic treatment. The test instrument used was the Empyrean X-ray diffractometer (PANalytical, Almelo, Netherlands). The tube voltage was 40 kV, the scanning angle range was 10°~50°, and the scanning step size was 2 °/s.

## 3. Results

### 3.1. AC Impedance and Ionic Conductivity

**Figure 6:**
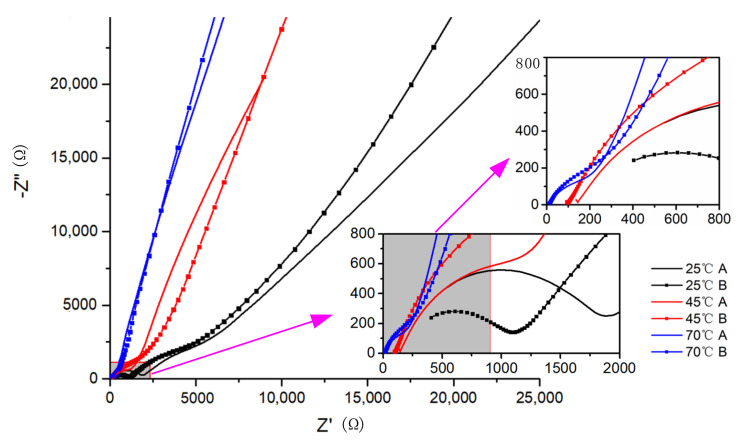
Alternating current (AC) impedance tests at different temperatures, where Group A is non-ultrasonic treated and Group B is ultrasonic treated.

The AC impedance spectra tested by the electrochemical workstation are shown in Figure 6. With the increase in the temperature, the impedance curves did not show a complete semicircle at low impedance region (high frequency), but gradually approached the oblique line at the diffusion region (low frequency). The rise in temperature promoted movement of the PEO chains, leading to the dominance of Li^+^ diffusion in the electrolyte. The impedance decreased gradually with the temperature rise, and the ionic conductivity increased. The melting temperature of the PEO is 65 °C. When the temperature was 70 °C, the electrolyte changed from the solid state to the molten one. PEO chains were released, and the impedance of the electrolyte was thus significantly reduced.

The ionic conductivity was derived from the AC impedance spectra. The ionic conductivity can be calculated by:

(1) $$\sigma =\frac{L}{R\times S}$$

where, R is the resistivity of the electrolyte membrane, S is the surface area, and L is the thickness. The membrane thickness was measured with a micrometer. 

The calculated ionic conductivity of the electrolytes from Group A and Group B is listed in Table 1.

**Figure 7:**
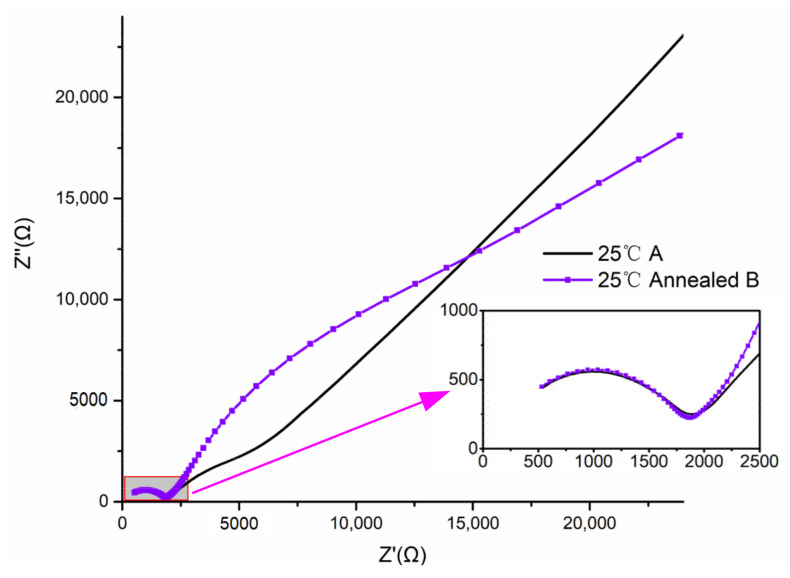
AC impedance test at 25 °C for the annealed electrolyte of Group B.

At 25 °C, the impedance of the electrolyte decreased from 1960 Ω to 1100 Ω due to the ultrasonic treatment, with a decrease of 44%. The corresponding ionic conductivity increased from 1.8 × 10^−6^ S/cm to 3.2 × 10^−6^ S/cm at 25 °C, and it was significantly improved by 78% with the ultrasonic treatment. At 45 °C, the ionic conductivity was also significantly improved due to the ultrasonic treatment. At 70 °C, the PEO-based electrolyte was molten. The chains were released completely, and thus the ultrasonic treatment had little effect on the electrolyte at this temperature, as shown in Figure 6. After the molten electrolyte was cooled to room temperature, it lost the increased conductivity completely. The impedance was tested for the annealed electrolyte of Group B, and the spectrum is shown in Figure 7. For comparison, the impedance spectrum of the electrolyte without the ultrasonic treatment (Group A) was also presented in the figure. The impedance measured 1942 Ω, and the corresponding ionic conductivity was 1.82 × 10^−6^ S/cm, which was similar to that of the electrolyte without the ultrasonic treatment (Group A of Table 1). 

### 3.2. Electronic Conductivity

**Figure 8:**
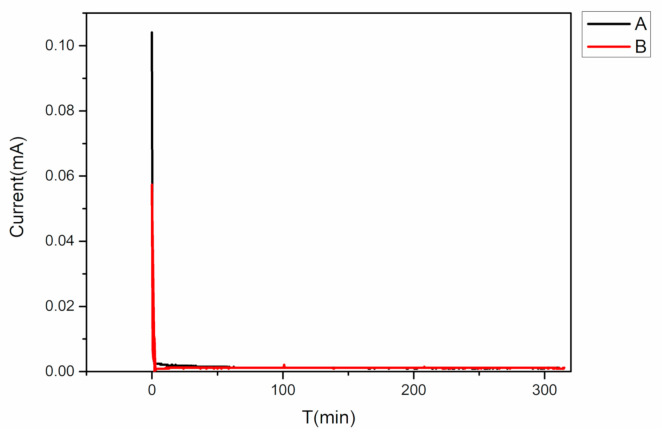
Direct Current (DC) polarization test results of the PEO-based solid electrolyte.

The DC polarization test results of the electrolytes are shown in Figure 8. It can be seen that the polarization current of the electrolyte without the ultrasonic treatment (Group A) was 0.103 mA, and the steady current was 0.0012 mA. The polarization current of the electrolyte treated by the ultrasonic vibration (Group B) was 0.0573 mA, and the steady current was 0.0012 mA. The results suggest that the ultrasonic treatment did not obviously change the electronic conductivity. The prepared electrolytes both had enough electronic resistivity. 

### 3.3. Electrochemical Window

**Figure 9:**
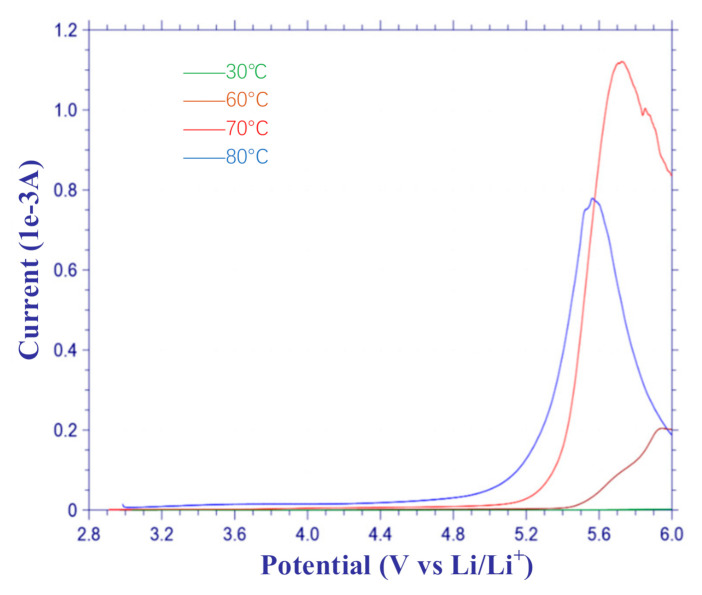
Linear sweep voltammetry (LSV) test results.

Electrolytes with a wide electrochemical window can match more types of electrode material. Therefore, a wide and stable electrochemical window is necessary for the electrolytes prepared. Currently, the electrochemical window of commercial lithium-ion batteries is 3~4 V. The electrochemical window of the prepared PEO-based electrolyte was tested by the LSV test, and the results are shown in Figure 9. The test result of Group A was similar to that of Group B, so we just presented that of Group B to illustrate the electrochemical window of the prepared electrolyte membrane.

As shown in Figure 9, the voltage threshold exceeded 6 V at 30 °C. As the voltage increased, the current of the electrolyte membrane did not change significantly until the voltage exceeded 5.0 V at 70 °C. With the temperature rising, the electrochemical window narrowed, and reached a minimum value of 4.8 V at 80 °C. This demonstrates that the prepared PEO-LiTFSI electrolyte membrane had a wide electrochemical window, which can satisfy the charge/discharge voltage of 2~3.6 V for lithium-ion batteries.

### 3.4. Mechanical Strength

**Figure 10:**
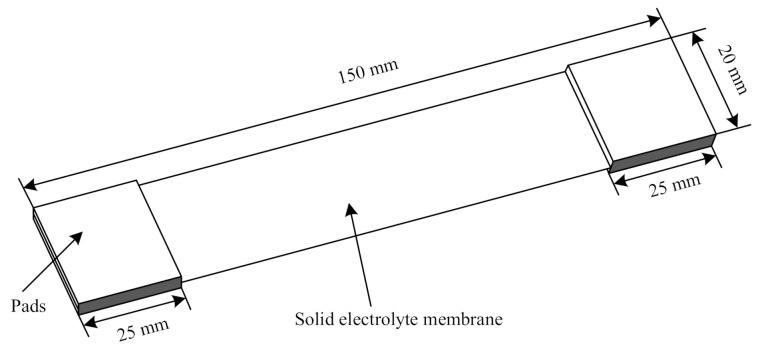
Sample of the electrolyte for the tensile test.

The mechanical strength of the electrolyte membrane is very important for all-solid-state lithium batteries. Sufficient mechanical strength can prevent short-circuiting caused by the penetration of lithium dendrite into the electrolyte membrane. The Instron134 material testing machine was used to test the tensile properties. The size of the sample is shown in Figure 10. The total length was 150 mm, the width was 20 mm, and the gauge length was 50 mm. The test was conducted at a speed of 5 mm/min. Two pads were added in the fixture area according to the standard of GB/T1040.3-2006, and the size was 25 × 20 × 1.5 mm.

**Figure 11:**
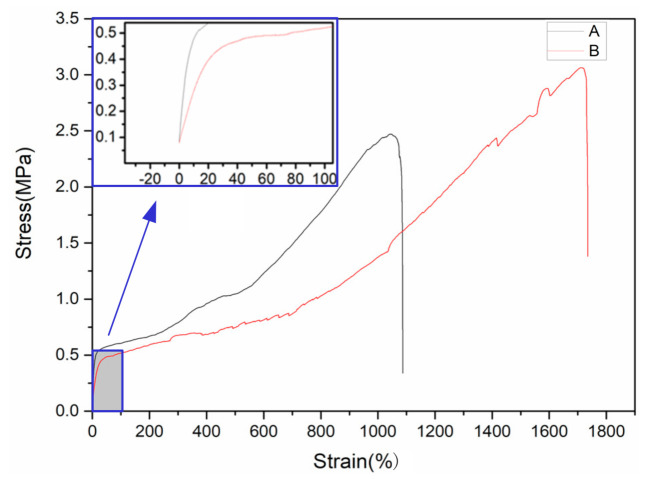
Stress–strain curves of the PEO-based electrolytes.

As shown in Figure 11, when the strain was below 20%, the tensile stress increased proportionally with the strain, indicating an elastic deformation. Furthermore, the curve of Group B had a significantly lower slope than that of Group A, which shows that the modulus of the electrolyte was decreased by the ultrasonic treatment. When the strain continued to increase, the stress increase rate slowed down, and the material underwent a plastic deformation. When the strain of Group A and Group B exceeded 600% and 800%, respectively, the stress rose rapidly. The polymer orientation of the electrolyte membranes increased, enhancing the material. Without the ultrasonic treatment (Group A), the electrolyte snapped at the strain of 1087%, and the tensile strength was 2.47 MPa. For the electrolyte treated by the ultrasonic process, the tensile failure occurred at the strain of 1734%, and the tensile strength was 3.06 MPa. The tensile strain and strength of the electrolyte was improved by the ultrasonic treatment. However, the yield strength of Group A was 0.6 MPa, while that of Group B was 0.49 MPa. The yield strength and elastic modulus of the electrolyte were decreased by the ultrasonic treatment.

### 3.5. Morphology

**Figure 12:**
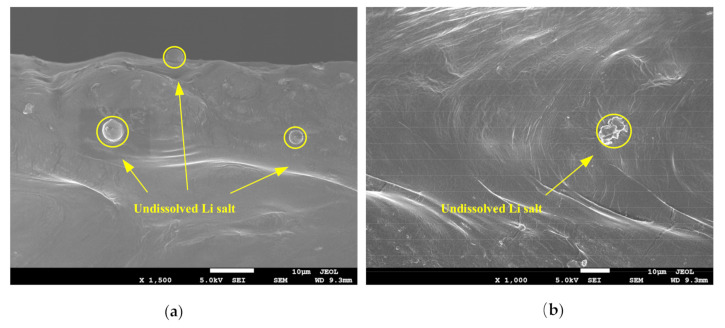
SEM observation of the electrolyte: (**a**) from Group A, and (**b**) from Group B.

The microstructure of the electrolyte membranes with and without the ultrasonic treatment was characterized by SEM. Figure 12 shows the cross-sectional morphology of the electrolytes. As shown in this figure, no obvious crack was observed inside the electrolyte membranes, indicating good toughness and low internal residual stress. Microparticles were observed inside the electrolytes, and were the undissolved lithium salt. As displayed in Figure 12, there were few particles, indicating that the prepared electrolytes were essentially homogeneous. Furthermore, the LiTFSI salt was evenly distributed, and thus the ratio of EO:Li=18:1 in the electrolytes was ensured, which was beneficial to the transport of Li^+^. Comparing Figure 12 a with b, material reflow lines were observed in the electrolyte membrane treated by the ultrasonic process. With the ultrasonic treatment, heat was generated in the electrolyte membrane. The ultrasonic vibration, increasing mobility of the polymer chains, was also beneficial to the material flow. At the same time, the membrane was compressed in the ultrasonic process, and thus the plastic flow of the material was produced, which modified the structure of the material. The solid electrolyte became tighter. In addition, we also measured the apparent density of the electrolyte. The density was 1.018 g/cm^3^ for the electrolyte of Group A, and was 1.136 g/cm^3^ for Group B. During the measurement, circular pieces of 10 mm in diameter were applied. The measured thickness was 0.081 mm and 0.072 mm for Group A and Group B, respectively, and the corresponding weight was 6.47 mg and 6.42 mg for them. From the measured density, the electrolyte with the ultrasonic treatment was tighter than that without the treatment. Therefore, the ion migration rate was increased by the ultrasonic treatment, and the mechanical strength was also improved.

### 3.6. Thermal Stability

**Figure 13:**
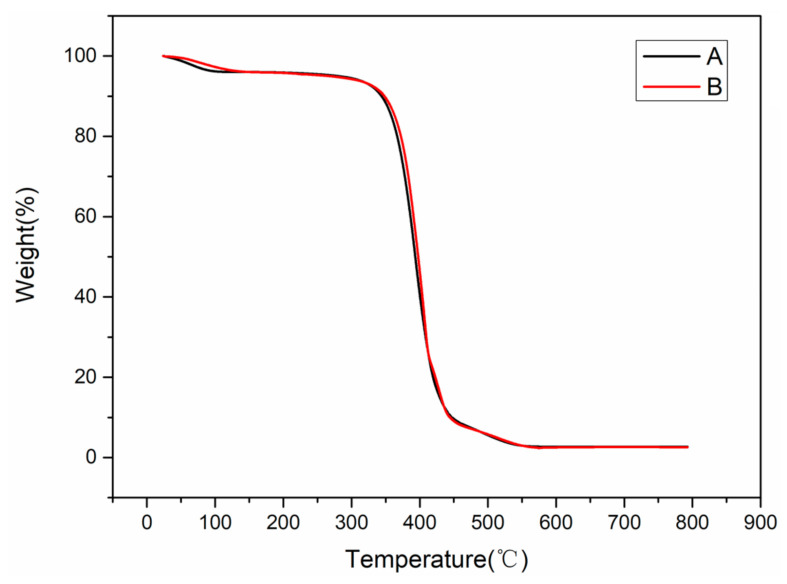
TGA results of the electrolytes.

The TGA test results are shown in Figure 13. The solid electrolyte membranes of both groups were thermally degraded within a temperature range of 300~400 °C, and the weight was lost primarily in one stage. The high temperature caused the degradation of PEO chains in the matrix. According to the FTIR spectrum analysis in Section 3.7, the initial mass loss was caused by the moisture absorption of the samples during the test. From the test data, the water loss was $1.428\;\times 10^{-5}$ mol for Group A, and $1.422\;\times 10^{-5}$ mol for Group B. The temperature of 150 °C was taken as the end point of the water lost. Furthermore, both samples began to decompose near 320 °C. This demonstrates that the ultrasonic treatment did not affect the thermal stability of the electrolyte membranes.

### 3.7. FTIR Analysis

**Figure 14:**
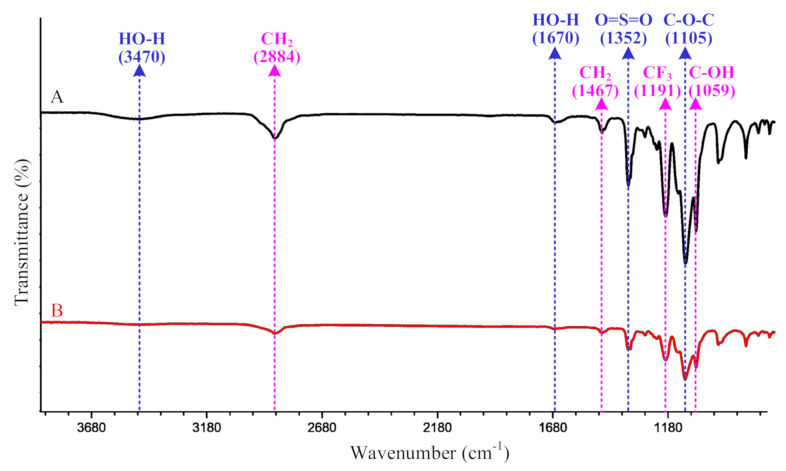
FTIR test results of the electrolytes.

The infrared spectra of the PEO-based electrolytes are shown in Figure 14. As displayed in this figure, the characteristic peaks were observed at 3470 cm^−1^ and 1670 cm^−1^ due to the water, which might be attributed to the absorbed moisture in the samples. Furthermore, no peak was observed near 2250 cm^−1^, the characteristic peak of C≡N in the acetonitrile, indicating that the residual solvent was not detected in the electrolyte. Therefore, the mass loss below 100 °C in Section 3.6 was due to evaporation of the absorbed moisture during the test.

Both spectra exhibited two peaks at 2884 cm^−1^ and 1467 cm^−1^, which were due to the stretching and bending vibration of CH_2_ in the PEO chains. The peak at 1352 cm^−1^ was due to the stretching vibration of the O=S=O group, while that at 1191 cm^−1^ was due to the stretching vibration of the –CF_3_ group. The two peaks were characteristic peaks of the LiTFSI salt. Ether group C–O–C in the PEO chains was identified at the characteristic peak of 1105 cm^−1^. By providing the electron-donor coordination site for the ion, the group C–O–C served as the function group for the Li^+^ ion transport because of the O atom. The end group C–OH in the PEO chains was also identified at the peak of 1059 cm^−1^. From the spectra in Figure 14, no obvious difference in the peak positions was found between the two groups, which demonstrated that the ultrasonic treatment did not alter the chemical composition of the solid electrolyte. On the other hand, the ultrasonic treatment did not exert a negative influence on the chemical composition by causing scission or degradation of the polymer chains.

### 3.8. Crystallinity

Crystallinity is the degree of structural order in the electrolyte. In a crystalline part, the molecules are arranged in a regular, periodic manner, and thus the crystallinity has a big influence on the migration of Li^+^.

**Figure 15:**
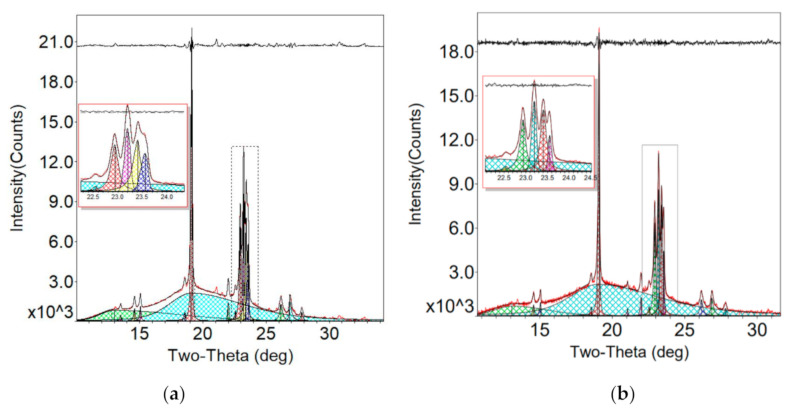
XRD analysis of the electrolytes from: (**a**) Group A, and (**b**) Group B.

For a highly crystallized material, crystalline grains tend to be intact. The XRD peak is strong and sharp, and the full width at half maxima (FWHM) is close to the instrumental broadening limit. For a poorly crystallized material, crystalline grains are smaller and fewer, and the defects are also increased. The diffraction peaks are broad and weak. In this study, XRD tests were performed on the electrolytes of both groups. The results were analyzed by Jade 6.0, as shown in Figure 15. As displayed in Figure 15, both spectra exhibited two low and broad peaks, indicating an amorphous phase. The 2θ values were 13.8° and 19.8°, respectively, and the FWHM was 8°. At 19° and 23°, sharp and narrow crystalline diffraction peaks appeared. Compared with the standard card, the diffraction peaks were the characteristic peaks of the PEO matrix. The simultaneous presences of the halo region and the crystalline peaks indicated the semi-crystalline nature of the PEO. The peak at 14.6° was due to LiTFSI in the electrolytes. 

As shown in Figure 15, the intensity of the PEO peaks was decreased for Group B due to the ultrasonic treatment, indicating a decrease in the PEO crystallinity and an increase in the PEO amorphous phase, which could enhance the ionic conductivity. 

From the XRD pattern, the crystallinity can be calculated by:

(2) $$X_{c}=\frac{I_{c}}{I_{t}}\times 100\%$$

where, $X_{c}$ is the crystallinity, and $I_{c}$ is the summed intensity of the crystalline peaks, and $I_{t}$ is of the total intensity. The calculated results show that the crystallinity of Group A (without the ultrasonic treatment) was 36.1%, and that of Group B (with the ultrasonic treatment) was 29.9%. In addition, with the ultrasonic treatment, the intensity of the crystalline peak of the PEO was significantly reduced at 19°. This demonstrated that due to the ultrasonic vibration treatment, the crystallinity of the electrolyte membrane was significantly reduced. The amorphous phase increased, which was beneficial to the transport of the lithium ions. The decrease in the crystallinity resulted from the mechanical and thermal effects of the ultrasonic high-frequency vibration. The mechanical effect broke the PEO crystalline grains, which decreased the grain size but increased the grain boundary. The high-frequency vibration caused friction at the grain boundary, and heat was generated there. Thus, the grains were melted from the boundary. Due to the pulse mode of the ultrasonic treatment, the melting was slight and the solidifying was fast. The crystallization was suppressed under this condition. Therefore, the crystallinity was reduced, improving the ionic conductivity of the electrolyte. On the other hand, as shown in the test result in Section 3.1, the electrolyte of Group B lost the increased conductivity after it was annealed to room temperature from a molten state. The morphology modified by the ultrasonic treatment was rearranged during the annealing process, and thus the effect of the ultrasonic treatment was eliminated. Furthermore, the lower crystallinity reduced the electrolyte yield strength but also improved the plasticity. From the XRD pattern, the amorphous peak was at 13.712° and 19.968° for the electrolyte of Group A, while the peak was at 13.851° and 20.268° for the electrolyte of Group B. The slight right shift of the amorphous peak illustrated the larger diffraction angle, indicating a denser structure. 

**Figure 16:**
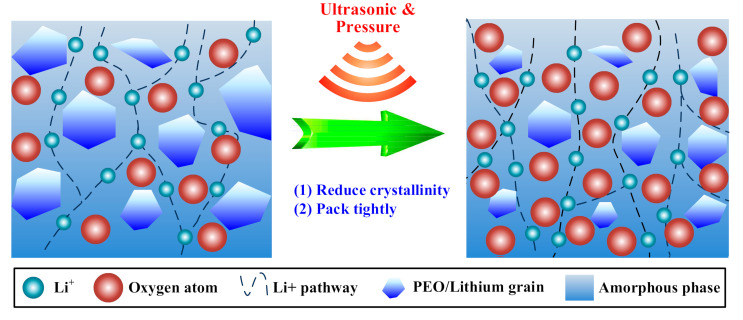
Ultrasonic promotion of ionic conductivity of the solid polymer electrolyte (SPE).

In the SPE, ethylene oxide (EO, the ether group) units have a high donor number for Li^+^ and high chain flexibility, which are important for promoting ion transport. The ion hopping mechanism is dominant in the ion transport [24]. The migration of Li^+^ from one site to the neighboring site is facilitated by the presence of vacancy defects, and therefore PEO crystallization is considered to be detrimental to ion transport due to the absence of vacancy in the area. According to the aforementioned analysis, the ultrasonic treatment brought large energy to the solid electrolyte through high-frequency vibration, which broke PEO grains and melted them with the frictional heat at the boundary. Due to the slight melting and fast solidifying produced by the pulse mode of the ultrasonic treatment, the crystallization was suppressed. Therefore, the treatment effectively reduced crystallinity of the PEO electrolyte membrane, which increased the migration channels of lithium ions. Smaller grains decreased the tortuosity effect as well, shortening the Li^+^ transport path, and thus improved the ionic conductivity. Moreover, the ultrasonic treatment compressed the electrolyte, inducing the plastic deformation, and thus the solid electrolyte became tighter. The density of EO units was increased in the amorphous phase, providing multiple electron-donor coordination sites for the Li^+^. The ion hopping distance between donors was decreased, which facilitated the migration. As a result, the ion migration rate was increased, as shown in Figure 16. In addition, the mechanical performance of the electrolyte membrane was thus improved.

## 4. Conclusions

In this study, the ultrasonic vibration method was used to improve the performance of the PEO-based solid electrolyte. The power ultrasound was exerted on the electrolyte by a sandwich structure. The ultrasonic treatment reduced the crystallinity of the electrolyte, and increased the proportion of the amorphous phase. Through analysis of the performance and microstructure of the electrolyte, the effect of the ultrasonic treatment on the electrolyte was studied. The main conclusions are as follows:

1. The ultrasonic treatment significantly improved the ionic conductivity of the PEO-based electrolyte. The ionic conductivity was increased by 78%. In addition, the ultrasonic treatment did not obviously affect the electronic conductivity. The prepared electrolyte had a wide electrochemical window, even at 80 °C.
2. The ultrasonic treatment broke PEO grains and melted them with the frictional heat at the boundary. Due to the slight melting and fast solidifying process, the crystallization was suppressed. The ultrasonic vibration effectively reduced the crystallinity by 6.2%, which increased the migration channels of lithium ions. Smaller grains decreased the tortuosity effect as well, shortening the Li^+^ transport path, and thus improved the ionic conductivity.
3. The ultrasonic treatment compressed the electrolyte to produce plastic flow of the material, which made the electrolyte structure more compact. The density of EO units was thus increased in the amorphous phase, providing multiple electron-donor coordination sites for the Li^+^. The hopping distance of the ion between donors was decreased as well, which also facilitated the migration.
4. The ultrasonic treatment did not alter the chemical composition and thermal stability of the solid electrolyte. The results show no negative effects produced, such as ultrasonic-induced bond break or thermal degradation.
5. The ultrasonic treatment improved the mechanical property of the electrolyte. The plasticity was improved, though the yield strength was slightly reduced. The tensile strength and elongation were both increased significantly.

This study presented a physical method to promote the ionic conductivity of the solid electrolyte using ultrasonic vibration, providing a reference for the improvement of polymer based all-solid-state batteries.

## Figures and Tables

**Table 1:** Impedance and ionic conductivity of the electrolytes.

	Impedance (Ω)		Ionic Conductivity (S/cm)	
	25 °C	45 °C	25 °C	45 °C
A	1960	144	1.8 × 10^−6^	2.4 × 10^−5^
B	1100	99	3.2 × 10^−6^	3.6 × 10^−5^
